# Engineering Kluyveromyces marxianus as a Robust Synthetic Biology Platform Host

**DOI:** 10.1128/mBio.01410-18

**Published:** 2018-09-25

**Authors:** Paul Cernak, Raissa Estrela, Snigdha Poddar, Jeffrey M. Skerker, Ya-Fang Cheng, Annika K. Carlson, Berling Chen, Victoria M. Glynn, Monique Furlan, Owen W. Ryan, Marie K. Donnelly, Adam P. Arkin, John W. Taylor, Jamie H. D. Cate

**Affiliations:** a Department of Molecular and Cell Biology, University of California, Berkeley, California, USA; b Innovative Genomics Institute, University of California, Berkeley, California, USA; c Department of Bioengineering, University of California, Berkeley, California, USA; d Biological Systems and Engineering Division, Lawrence Berkeley National Laboratory, Berkeley, California, USA; e Energy Biosciences Institute, Berkeley, California, USA; f Department of Chemistry, University of California, Berkeley, California, USA; g Department of Environmental Science, Policy, and Management, University of California, Berkeley, California, USA; h Department of Genetics, Evolution and Bioagents, University of Campinas, Campinas, São Paulo, Brazil; i Department of Plant and Microbial Biology, University of California, Berkeley, California, USA; j Environmental Genomics and Systems Biology Division, Lawrence Berkeley National Laboratory, Berkeley, California, USA; k Molecular Biophysics and Integrated Bioimaging, Lawrence Berkeley National Laboratory, Berkeley, California, USA; Korea Advanced Institute of Science and Technology

**Keywords:** CRISPR-Cas9, *Kluyveromyces marxianus*, lipogenesis, mating, renewable chemicals, thermotolerant yeast

## Abstract

Throughout history, the yeast Saccharomyces cerevisiae has played a central role in human society due to its use in food production and more recently as a major industrial and model microorganism, because of the many genetic and genomic tools available to probe its biology. However, S. cerevisiae has proven difficult to engineer to expand the carbon sources it can utilize, the products it can make, and the harsh conditions it can tolerate in industrial applications. Other yeasts that could solve many of these problems remain difficult to manipulate genetically. Here, we engineered the thermotolerant yeast Kluyveromyces marxianus to create a new synthetic biology platform. Using CRISPR-Cas9 (clustered regularly interspaced short palindromic repeats with Cas9)-mediated genome editing, we show that wild isolates of K. marxianus can be made heterothallic for sexual crossing. By breeding two of these mating-type engineered K. marxianus strains, we combined three complex traits—thermotolerance, lipid production, and facile transformation with exogenous DNA—into a single host. The ability to cross K. marxianus strains with relative ease, together with CRISPR-Cas9 genome editing, should enable engineering of K. marxianus isolates with promising lipid production at temperatures far exceeding those of other fungi under development for industrial applications. These results establish K. marxianus as a synthetic biology platform comparable to S. cerevisiae, with naturally more robust traits that hold potential for the industrial production of renewable chemicals.

## Abstract (precis)

The yeast Kluyveromyces marxianus grows at high temperatures and on a wide range of carbon sources, making it a promising host for industrial biotechnology to produce renewable chemicals from plant biomass feedstocks. However, major genetic engineering limitations have kept this yeast from replacing the commonly used yeast Saccharomyces cerevisiae in industrial applications. Here, we describe genetic tools for genome editing and breeding K. marxianus strains, which we use to create a new thermotolerant strain with promising fatty acid production. These results open the door to using K. marxianus as a versatile synthetic biology platform organism for industrial applications.

## INTRODUCTION

Synthetic biology is used to harness the metabolic capacity of microorganisms for the biosynthesis of simple and complex compounds now sourced unsustainably from fossil fuels or that are too expensive to make using chemical synthesis at industrial scale. The yeast Saccharomyces cerevisiae has served as the major eukaryotic organism for synthetic biology, but lacks the metabolic potential that could be exploited in many of the more than one thousand yeast species that have been identified to date. These yeasts remain difficult to use, however, as there are few synthetic biology tools to access their underlying metabolic networks and physiology (1). The budding yeast Kluyveromyces marxianus possesses a number of beneficial traits that make it a promising alternative to S. cerevisiae. K. marxianus is the fastest-growing eukaryotic organism known (2), is thermotolerant, growing and fermenting at temperatures up to 52 and 45°C, respectively (3), and uses a broad range of carbon sources, including pentose sugars. These traits are polygenic and would be difficult to engineer into a less robust host such as S. cerevisiae. K. marxianus also harbors high strain-to-strain physiological and metabolic diversity, which could prove advantageous for combining beneficial traits by sexual crossing. However, K. marxianus is generally found to be homothallic (4, 5) (i.e., is self-fertile) and cannot be crossed in a controlled manner.

For K. marxianus to be useful as a yeast platform for synthetic biology, it will be essential to establish efficient gene editing tools along with methods to cross strains with stable ploidy. These tools would enable rapid strain development, by generating genetic diversity and facilitating stacking of industrially important traits. The CRISPR-Cas9 (clustered regularly interspaced short palindromic repeats with Cas9) gene editing system has been used in many yeasts, including S. cerevisiae, Schizosaccharomyces pombe, Yarrowia lipolytica, Kluyveromyces lactis, and recently K. marxianus (6–9). Genome editing in K. marxianus should allow manipulation of known genetic targets. However, most desired traits likely depend on multiple, unlinked genetic loci, which remain difficult to identify without the ability to carry out genetic crosses. The ability to cross phenotypically diverse S. cerevisiae strains has been an indispensable tool for exploring its biology on a genome-wide scale and for improving its use as an industrial host (10). To approach the versatility of S. cerevisiae genetics, it will be necessary to gain full control over K. marxianus ploidy and mating type. As a homothallic yeast, K. marxianus lacks a permanent mating type, because K. marxianus haploid cells naturally change their mating type (either the **a** mating type, *MAT***a**, or α mating type, *MAT*α) leading to uncontrolled *MAT***a**/*MAT*α diploidization within a population (11). This stochastic ploidy makes it impossible to carry out quantitative biological studies of interesting traits that are ploidy specific (12), as it leads to populations with mixed phenotypes and prevents K. marxianus domestication through selective crossing.

To overcome the limitations in using K. marxianus as a synthetic biology platform, we adapted the CRISPR-Cas9 system we developed for S. cerevisiae (13) for use in K. marxianus, enabling both nonhomologous end joining (NHEJ) and homology-directed repair (HDR)-based genome editing. We identified the genetic loci responsible for mating-type switching in K. marxianus and created domesticated laboratory strains by simultaneously inactivating these genes. With this platform in place, we explored a large collection of wild K. marxianus strains to investigate K. marxianus lipid production at high temperatures. By domesticating and crossing promising strains, we were able to combine three complex traits—the ability to take up exogenous DNA (transformability), thermotolerance, and higher lipid production—into single K. marxianus isolates.

## RESULTS

### CRISPR-Cas9 system in K. marxianus.

**FIG 1:**
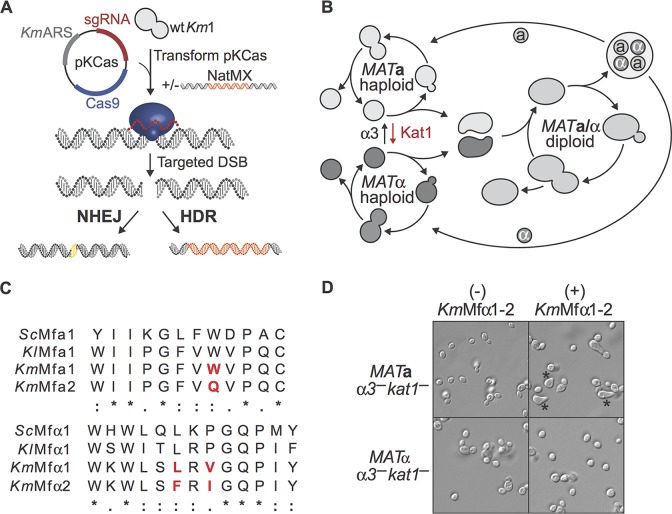
CRISPR-Cas9 genome editing and mating-type switching in K. marxianus. (A) CRISPR_NHEJ_ and CRISPR_HDR_ systems. K. marxianus transformed with the pKCas plasmid generates small indels near the cut site, a common product of nonhomologous end joining (NHEJ) repair of the DNA double-strand break. When transformed with both the pKCas plasmid and a donor DNA, homologous recombination products are seen in the target site. (B) Yeast life cycle. Haploid *MAT***a** and *MAT*α switch mating type by transposases α3 and Kat1 in K. lactis. Haploid cells conjugate to form *MAT***a**/*MAT*α diploids. Diploids undergo meiosis to form haploid spores that germinate to complete the life cycle. (C) Mature **a**- and α-pheromones from K. marxianus aligned with the S. cerevisiae and K. lactis sequences. Red indicates nonconserved amino acids between K. marxianus **a**- and α-factors. Amino acids are marked as identical (*), with similar polarity (:), or with different polarity (.). (D) Incubation of putative heterothallic *MAT***a** and *MAT*α strains with a cocktail of both mature α-factor pheromones (*Km*Mfα1 and -2) results in mating projections from the *MAT***a** strain only (*).

We established robust genome editing in K. marxianus by adapting the plasmid-based CRISPR-Cas9 (CRISPRm) system we previously developed for S. cerevisiae (13). We first identified a K. marxianus-specific origin of replication and K. marxianus-specific promoters and terminators for expressing Cas9 (see Materials and Methods). We used the S. cerevisiae gene for tRNA^Phe^ as an RNA polymerase III promoter to express single-guide RNAs (sgRNAs) from the same plasmid (Fig. 1A). To test the effectiveness of the redesigned CRISPR-Cas9 system, we used a wild strain we isolated from a sugarcane bagasse pile (Km1 [Table 1]), and a Km1-derived *MAT***a** heterothallic strain (Km30 [see Table S1 in the supplemental material]). We transformed the pKCas plasmid (G418^R^) carrying an sgRNA targeting the *URA3* gene into Km1, plated on G418 plates, and then selected for 5-fluoroorotic acid (5-FOA) resistance by replica plating to identify *ura3*^–^ colonies. The efficiency of NHEJ-based Cas9 editing (CRISPR_NHEJ_)—the number of *ura3^–^* colonies divided by the number of total G418^R^ transformants—was near 90% and was confirmed by sequencing the *URA3* locus. Targeting other genes across the genome resulted in around 75% efficiency (see Fig. S1 in the supplemental material). To test the ability of the K. marxianus CRISPR system to insert exogenous DNA at a defined locus, we also cotransformed into strain Km30 the pKCas plasmid encoding a guide RNA targeting the *URA3* gene along with a double-stranded DNA repair template comprised of a linear nourseothricin resistance cassette flanked by K. marxianus *URA3* homology sequences adjacent to the Cas9 target site (NatMX flanked by 0.9-kb homology arms). Using replica plating of G418^R^ transformants onto two selection plates—5-FOA to detect *ura3^–^* alleles and Nat^R^ to detect HDR events—we found 100% of the colonies to be *ura3^–^* and ∼97% (189/195) to be Nat^R^, indicating that repair of Cas9-induced double-strand breaks allowed highly efficient HDR-mediated gene integration (CRISPR_HDR_). We used colony PCR on select Nat^R^ colonies (targeting outside the 0.9-kb homology arms of the NatMX cassette) to confirm NatMX cassette integration at the *URA3* locus.

**TABLE 1:** List of wild-type K. marxianus strains used in this work

Strain^*a*^	Designation no. by:			
	ATCC	NCYC	CBS	NRRL
Km1				
Km2	10022	100	6432	Y-665
Km5	46537	851	397	Y-2415
Km6		143	608	Y-8281
Km9	26548	2597	6556	Y-7571
Km11	36907	587		
Km16	10606		396	Y-1550
Km17	8635/28910			Y-1190
Km18			1089	Y-2265
Km19	26348			
Km20				
Km21				

a Km1, Km20, and Km21 were isolated from sugarcane bagasse piles. Km1 was isolated at Raceland Raw Sugar Corporation, Raceland, LA. Km20 and Km21 were isolated at the Sugarcane Growers’ Cooperative, Belle Glade, FL.

10.1128/mBio.01410-18.1 FIG S1 Cas9 editing outcomes and efficiency for several K. marxianus genes. (A) CRISPR_NHEJ_ results for *KmURA3*-targeting experiments in the absence of donor DNA. Three regions were targeted, and small insertions and deletions were found upon repair of double-strand breaks by the NHEJ machinery. (B) Small indels can also be observed when targeting *KmKAT1* and *KmALPHA3* genes. (C) Editing efficiency across 10 different K. marxianus genes is around 75%, with some genes more editing prone than others. Download FIG S1, TIF file, 2.66 MB. Copyright © 2018 Cernak et al. 2018 Cernak et al. This content is distributed under the terms of the Creative Commons Attribution 4.0 International license.

10.1128/mBio.01410-18.8 TABLE S1 List of engineered K. marxianus strains. Download Table S1, XLSX file, 0.04 MB. Copyright © 2018 Cernak et al. 2018 Cernak et al. This content is distributed under the terms of the Creative Commons Attribution 4.0 International license.

### Engineering mating-competent heterothallic K. marxianus strains.

We used CRISPR_NHEJ_ to make stable K. marxianus laboratory strains with defined ploidy and mating type to enable the use of classical yeast genetics. Most naturally isolated K. marxianus strains are homothallic: i.e., they change their mating type spontaneously by “mating-type switching” to create mixed populations of *MAT***a**, *MAT*α, and *MAT***a**/*MAT*α cells (4, 5). The K. marxianus mating-type switching mechanism is not genetically conserved with the well-characterized HO endonuclease mechanism employed by S. cerevisiae. Notably, a two-component switching mechanism has been identified in Kluyveromyces lactis (14, 15), which uses two transposases (Kat1 and α3) for *MAT* switching. The α3 transposase switches *MAT*α type cells to *MAT***a** type, and Kat1 switches *MAT***a** to *MAT*α type (Fig. 1B).

We identified the K. marxianus orthologs of the K. lactis *KAT1* and *ALPHA3* genes using reciprocal BLASTp against predicted open reading frames (ORFs) from the whole-genome sequence of Km1 (see Table S2 in the supplemental material) (16). Using CRISPR_NHEJ_, we targeted both transposase genes (Table S2) to create frameshift mutation loss-of-function alleles. We then isolated several of these double-transposase-inactivated Km1 *α3^–^ kat1^–^* strains that had small base pair insertions or deletions near the Cas9 cut site (Fig. S1). To identify *MAT***a** haploid isolates, we used a pheromone morphological response assay. Yeast mating is initiated by the secretion of small peptide pheromones **a**-factor and α-factor by *MAT***a** and *MAT*α cells, respectively. The pheromones, derived from **a**-pheromone and α-pheromone precursor proteins, mating factor **a** (MFA1 and MFA2) and mating factor α (MFα1), are detected by their cognate cell surface recognition proteins and lead to polar morphogenesis or the formation of mating projections (“shmoo”) that can be used to deduce a strain’s mating type. We identified two putative K. marxianus *MFA* genes (*KmMFA1* and *KmMFA2*) as well as the *MF*α gene (*KmMFα1*, encoding two isotypes, *Km*MFα1 and *Km*MFα2) in the K. marxianus genome by reciprocal BLASTp using the S. cerevisiae and K. lactis protein sequences as queries (16, 17) (Fig. 1C; see Fig. S2A in the supplemental material). Incubation of K. marxianus strain Km1 *α3^–^ kat1^–^* cells with synthetic *Km*MFα1 and *Km*MFα2 peptide pheromones resulted in isolates that responded to both α-factors (Fig. 1D), indicating these are *MAT***a** α*3^–^ kat1^–^* haploids. We categorized unresponsive strains as either *MAT*α or diploid strains, using sequencing of the *MAT* locus (Fig. S2B).

10.1128/mBio.01410-18.2 FIG S2 K. marxianus mating. (A) K. marxianus **a**- and α-factor protein sequences. Mature pheromones (in red) derive from precursor proteins, mating factor ****a**** (MFA1 and MFA2) and mating factor α (MFα1). We identified two putative *MFA* genes as well as the *MF*α gene (*KmMFα1* encoding 2 isotypes, *Km*MFα1 and *Km*MFα2) in the K. marxianus genome by reciprocal BLASTp using the S. cerevisiae and K. lactis protein sequences as queries. (B) Proposed architecture of K. marxianus *MAT***a**, *MAT*α, *HMR***a**, and *HMLα* loci. Black arrows indicate annealing sites for the genotyping primers (Table S2). PCR of an **a**-type strain yielded an ∼3,480-bp product, while the α-type yielded an ∼6,500-bp PCR product. Download FIG S2, TIF file, 0.56 MB. Copyright © 2018 Cernak et al. 2018 Cernak et al. This content is distributed under the terms of the Creative Commons Attribution 4.0 International license.

10.1128/mBio.01410-18.9 TABLE S2 DNA sequences for sgRNA spacers, primers, and genes. Download Table S2, XLSX file, 0.05 MB. Copyright © 2018 Cernak et al. 2018 Cernak et al. This content is distributed under the terms of the Creative Commons Attribution 4.0 International license.

**FIG 2:**
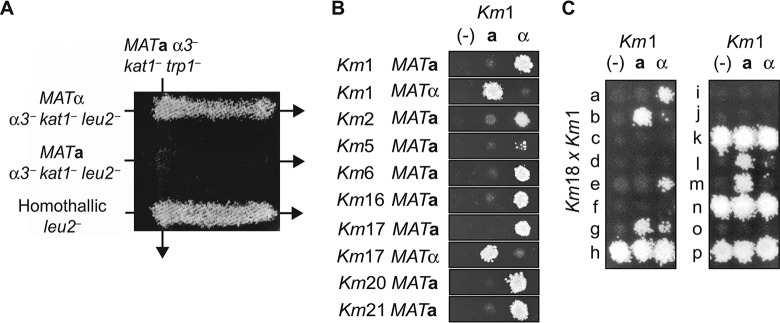
Creation of heterothallic K. marxianus strains. (A) Auxotrophic mating assay of Km1 strains. Shown are results from strains Km1 *MAT*α *α3^–^ kat1^–^ leu2^–^*, Km1 *MAT***a** *α3^–^ kat1^–^ leu2^–^*, and homothallic Km1 *leu2*^–^, streaked through strain Km1 *MAT***a** *α3^–^ kat1^–^ trp1^–^* on 2% glucose plates and replica plated onto SCD − (Leu, Trp) plates after 2 days. Diploid growth is seen only upon sexual crossing between strains with opposite mating types or with homothallic haploid strains. (B) Auxotrophic mating assay of several *α3^–^ kat1^–^ leu2^–^* triple*-*inactivation strains and Km1 *MAT***a** *α3^–^ kat1^–^ trp1^–^* or Km1 *MAT*α *α3^–^ kat1^–^ trp1^–^*. Putative heterothallic strains were spotted over the negative control (−), the Km1 *MAT***a** *α3^–^ kat1^–^ trp1^–^* reference (**a**), or the Km1 *MAT*α *α3^–^ kat1^–^ trp1^–^* reference (α) on glucose plates for mating. Replica plating onto SCD − (Leu, Trp) results in diploid growth. (C) The wild homothallic isolate Km18 was made *trp^–^* by UV mutagenesis and crossed with heterothallic *Km*1 *MAT***a** *α3^–^ kat1^–^ leu2^–^*. Diploids were sporulated, 16 spores were isolated (a through p) and germinated, and the resulting haploids were screened for heterothallic strains by crossing with Km1 *MAT***a** *α3^–^ kat1^–^ trp1^–^* or Km1 *MAT*α *α3^–^ kat1^–^ trp1^–^*. Screened haploids were auxotrophic strains unable to mate (c, d, f, i, j, and o), possible *trp^–^* revertants (h, k, n, and p), homothallic (g), or heterothallic (a, b, e, l, and m).

Once we identified stable *MAT***a** α*3^–^ kat1^–^* or *MAT*α α*3^–^ kat1^–^* strains through the pheromone response method and subsequent sequencing of the *MAT* locus (Table S2 and Fig. S2B), we tested for the ability of two haploid strains with different auxotrophic markers to mate and form prototrophic diploids. First, we used CRISPR_NHEJ_ to create *leu2*^–^ or *trp1*^–^ auxotrophic mutants of the predicted heterothallic *MAT***a** α*3*^–^ *kat1^–^* and *MAT*α α*3^–^ kat1^–^* or *MAT***a**/*MAT*α strains. Then, *MAT***a** α*3^–^ kat1^–^ trp1*^–^ and *MAT*α α*3^–^ kat1^–^ leu2*^–^ strains were combined by opposing streaks on mating-inducing medium. After 2 days, successful mating of *MAT***a** α*3^–^ kat1^–^ trp1*^–^ and *MAT*α α*3^–^ kat1^–^ leu2*^–^ cells resulted in growth when cells were replica plated onto minimal medium lacking tryptophan and leucine (Fig. 2A). To test whether the engineered heterothallic strains could still mate with homothallic wild-type isolates, heterothallic Km1 *MAT***a** α*3^–^ kat1^–^ trp1*^–^ cells were streaked with the homothallic haploid Km1 *leu2^–^* strain, resulting in mating and diploid growth (Fig. 2A). These data confirm the CRISPR_NHEJ_-engineered Km *α3^–^ kat1^–^* strains are heterothallic and result in stable haploid, breeding-competent isolates that can mate with opposite mating types and wild homothallic strains.

To further validate the role of α3 and Kat1 in switching mating types, we performed complementation assays by constitutively expressing these transposases from plasmids. Plasmids encoding Kat1 or α3 were transformed into Km1 *α3^–^ kat1^–^ leu2^–^* strains to revert the controlled mating phenotype and promote homothallism. Transformants were then tested for mating-type switching by crossing them with stable heterothallic reference strains (*α3^–^ kat1^–^ trp1^–^*) of either *MAT***a** or *MAT*α mating type. Using the auxotrophic mating assay, these experiments showed that complementing stable *MAT***a** mutants with Kat1 overexpression plasmids or stable *MAT*α mutants with α3-expressing plasmids induced mating-type switching. Kat1 caused *MAT***a** isolates to switch and mate with a *MAT***a** reference strain, and α3 caused *MAT*α isolates to switch and mate with a *MAT*α reference strain (see Fig. S3 in the supplemental material).

10.1128/mBio.01410-18.3 FIG S3 Kat1 and α3 rescue of mating-type switching. Plasmid-based ectopic expression of Kat1 and α3 restores mating-type switching in *MAT***a** *α3^*–*^ kat1^*–*^* and *MAT*α *α3^*–*^ kat1*^–^, respectively. SCD − (Leu, Trp) plates are shown where only diploid strains are able to grow. (A) Ectopic expression of Kat1 allowed a stable *MAT***a** *α3^*–*^ kat1^*–*^ leu2^*–*^* strain to switch to *MAT*α and mate with the *MAT***a** *α3^*–*^ kat1^*–*^ trp1^*–*^* reference strain. (B) Similarly, ectopic expression of α3 allowed some *MAT*α transformants to switch to *MAT***a** and mate with the *MAT*α reference strain. Note that some *MAT*α transformants did not switch to *MAT***a**, keeping the ability to mate with *MAT***a**. Download FIG S3, TIF file, 1.70 MB. Copyright © 2018 Cernak et al. 2018 Cernak et al. This content is distributed under the terms of the Creative Commons Attribution 4.0 International license.

A strong advantage of turning K. marxianus into a synthetic biology chassis for metabolic engineering is its high strain-to-strain phenotypic and metabolic diversity (18). To build a widely useful yeast platform, we sought to create heterothallic strains of each mating type for 12 wild isolates collected from the ATCC and CBS culture collections and our own isolates (Table 1). These strains have been isolated from diverse locations and substrates around the world, from dairy to sugarcane bagasse. We used CRISPR_NHEJ_ to create strains with inactivation of three genes: those encoding the transposases Kat1 and α3 and an auxotrophic marker, either *TRP1* or *LEU2.* Triple-inactivation strains (*α3^–^ kat1^–^ leu2^–^* or *α3^–^ kat1^–^ trp1^–^*) were successfully isolated from 10 of the isolates. We assayed these strains for mating type by crossing them with heterothallic Km1 strains as a reference, using the auxotrophic mating assay described above. Heterothallic haploids (*MAT***a** and/or *MAT*α) were isolated from 10 of the triple-inactivation strains (Fig. 2B). For strain Km18, which was difficult to transform with plasmid DNA, stable heterothallic strains could be isolated from a cross between a homothallic Km18 strain first made *trp^–^* using UV mutagenesis and Km1 heterothallic strains. The Km18 *trp^–^* × Km1 diploids were sporulated and germinated and then back-crossed with Km1 haploid reference strains to establish their mating type (Fig. 2C).

### K. marxianus strains engineered for higher levels of lipogenesis.

**FIG 3:**
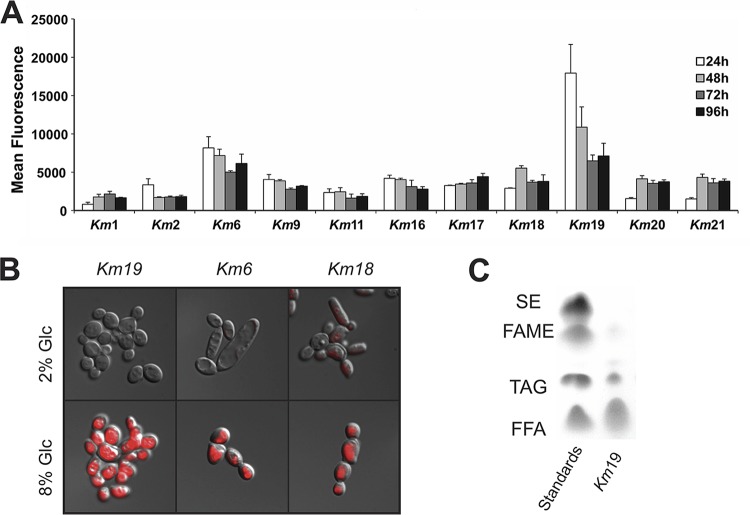
Lipogenesis of K. marxianus strains. (A) Nile red fluorescence flow cytometry of 11 wild-type isolates after 24, 48, 72, and 96 h at 42°C in lipogenesis medium. Experiments were carried out in biological triplicate, with means and standard deviations shown. (B) DIC images superimposed with epifluorescence microscopy of Nile red-stained cells. Little or no fluorescence is seen after 24 h in 2% glucose. After 24 h in 8% glucose at 42°C (Km19 and Km6) and 48 h (Km18), fluorescence is seen encompassing the majority of the cell volume. (C) TLC analysis of Km19 total lipids after 24 h in 8% glucose at 42°C. Lane 1, ladder of standards containing steryl ester (SE), fatty acid methyl ester (FAME), triacylglycerols (TAG), and free fatty acids (FFA). Lane 2, Km19 lipids.

To explore the industrial potential of K. marxianus compared to S. cerevisiae (i.e., thermotolerance and Crabtree-negative growth, preferring respiration over fermentation [19]), we tested lipid production in K. marxianus under aerobic conditions. We first screened 11 wild-type K. marxianus isolates for levels of lipogenesis using a lipophilic fluorescent dye (Nile red), combined with flow cytometry and cell sorting. Nile red localizes to lipid droplets in yeast and exhibits increased red fluorescence proportional to the total amount of lipid in the cell (20, 21). The K. marxianus strains were grown in 8% glucose or 8% cellobiose lipogenesis medium at 30 and 42°C, and time point samples were collected every 24 h to be analyzed by flow cytometry. The highest fluorescence was observed in strains fed 8% glucose at 42°C for 24 h, with large strain-to-strain differences spanning an ∼20-fold change in fluorescence (Fig. 3A). A few strains also produced significant amounts of lipid in cellobiose at 42°C, compared to their production of lipid in glucose (i.e., strains Km2 and Km17 [Table 1]) (see Fig. S4A in the supplemental material). All strains produced much lower levels of lipid when grown at 30°C.

10.1128/mBio.01410-18.4 FIG S4 Lipogenesis of K. marxianus strains. (A) Strains grown on 8% cellobiose. Shown is mean Nile red fluorescence flow cytometry of 11 wild-type isolates after 24, 48, 72, and 96 h at 42°C in lipogenesis medium containing 8% cellobiose. Maximum values do not surpass 30% of the value obtained for the top lipid-producing strain grown on glucose. Experiments were carried out in biological triplicate, with means and standard deviations shown. (B) Lipid accumulation in K. marxianus strains. Shown are Km19, Km17, and Km6 percentages of fatty acids in dry cell weight after 24 h in 8% glucose at 42°C and 250 rpm. Lipogenesis medium contained ammonium sulfate instead of monosodium glutamate for this set of measurements. Measurements were from biological triplicates, with mean and standard deviation shown. Download FIG S4, TIF file, 0.35 MB. Copyright © 2018 Cernak et al. 2018 Cernak et al. This content is distributed under the terms of the Creative Commons Attribution 4.0 International license.

We used fluorescence microscopy to examine the cell morphology of the strains with the highest lipid titers. When Nile red fluorescence was overlaid with differential interference contrast (DIC) images of K. marxianus isolates Km19, Km6, and Km18 (Table 1) after 24 or 48 h of growth in 8% glucose at 42°C, large lipid droplets encompassed a large fraction of the cell volume (Fig. 3B). Km19 produced the highest levels of lipids as measured by Nile red fluorescence, which peaked after only 24 h (Fig. 3A), at which point Km19 had accumulated lipids at ∼10% dry cell weight (Fig. S4B). Thin-layer chromatography (TLC) revealed that the majority of the lipid in Km19 accumulated as free fatty acids (FFAs) (Fig. 3C).

### Strain engineering for higher lipid production.

**FIG 4:**
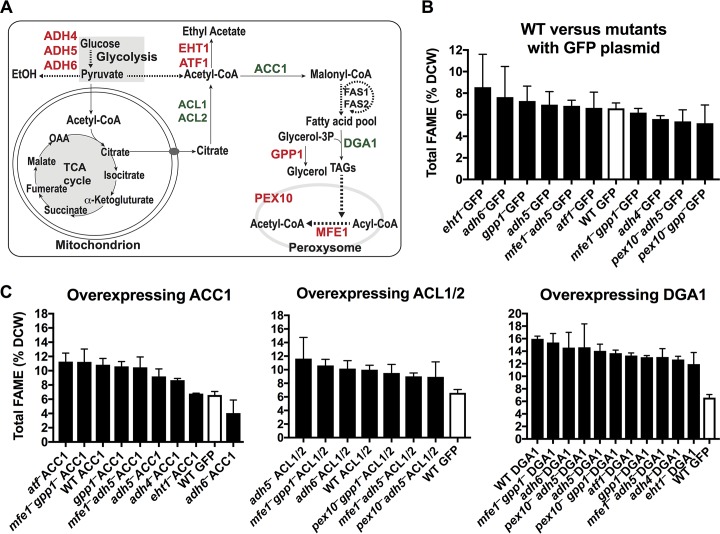
Genetic dissection of lipogenesis of a high-producing K. marxianus strain, Km6. (A) General overview of lipid-related metabolism. Genes in red were inactivated with CRISPR_NHEJ_, and genes in green were overexpressed using plasmids. (B) Percentage of fatty acids in dry cell weight (DCW) after 24 h under lipogenic conditions at 42°C for several variants of Km6 (wild type and mutants). (C) Percentage of fatty acids in dry cell weight for several Km6 variants containing *ACC1*, *DGA1*, and *ACL1/2* overexpression plasmids. In panels B and C, all experiments were carried out in biological triplicate, with mean values and standard deviations shown. Lipogenesis medium in panels B and C contained monosodium glutamate instead of ammonium sulfate.

Lipogenesis in oleaginous yeasts such as Yarrowia lipolytica results in the synthesis and storage of lipid droplets within the cytoplasm (22). Lipid biosynthesis is largely dependent upon the enzymes AMP deaminase (AMPD), ATP-citrate lyase (ACL), acetyl coenzyme A (acetyl-CoA) carboxylase (ACC), and malic enzyme (MAE) (23). Collectively, these enzymes promote the accumulation of acetyl-CoA via citrate. Interestingly, although ACL is thought to be crucial for lipogenesis in oleaginous yeasts (24), we did not identify the genes *ACL1* and *ACL2* in the K. marxianus reference strain, Km1. ACC1 then converts acetyl-CoA into malonyl-CoA, and the malic enzyme provides NADPH, the reduced cofactor necessary for the production of lipids. Total lipid accumulation is a balance between lipid synthesis and catabolism through β-oxidation in the peroxisome (Fig. 4A). Engineered strains of Y. lipolytica with reduced β-oxidation (*pex10*Δ) and peroxisome biogenesis (*mfe1*Δ) combined with overexpression of lipogenesis enzymes can store up to 80 to 90% dry cell weight as lipid compared to only ∼10 to 15% lipid content for wild-type cells (23). However, the high yield of lipogenesis in Y. lipolytica often takes up to 5 days to reach its peak (25) and requires temperatures of ∼30°C, due to the lack of thermotolerance in this yeast (23).

We tested whether inactivation or overexpression of genes previously shown in Y. lipolytica to contribute to high lipid production (23) would affect the ability of K. marxianus to produce lipids. Although wild-type Km19 produced the most lipids of the wild-type strains we tested, Km19 is very difficult to transform with plasmids. Therefore, we chose to use strain Km6 (Table 1), since it has similar lipid content and is easily transformed with plasmid DNA, allowing the facile use of plasmid-based CRISPR-Cas9 to inactivate genes or plasmid-based overexpression.

Unlike Y. lipolytica, fermentation of glucose to ethanol and esterification of acetate to ethyl acetate are likely to compete with lipogenesis in K. marxianus. Therefore, we inactivated genes by CRISPR_NHEJ_ to decrease ethanol fermentation (*ADH* genes) (26) and ethyl acetate production (*ATF* genes) (7), as well as ester biosynthesis (*EHT1*) (7, 27) and glycerol biosynthesis (*GPP1*). We also inactivated genes involved in β-oxidation (*PEX10* and *MFE1*) (23) (Fig. 4A). For the overexpression experiments, we cloned genes known to be involved in the accumulation of lipids (*DGA1*, *ACC1*, and the dimer *ACL1*/*ACL2*) into overexpression plasmids constructed using strong promoters (*KmTDH3* or *KmPGK1*) to drive the expression of these genes. The plasmids were individually transformed into wild-type Km6 or strains in which CRISPR_NHEJ_ had been used for targeted gene inactivation. The fatty acid content of each of these engineered strains was measured by gas chromatography and calculated as the percentage of dry cell weight. Although none of the inactivated genes had an appreciable effect on the accumulation of fatty acids (Fig. 4B), overexpression of *DGA1* increased the levels of fatty acids across all strains tested, more than doubling the fatty acid content in the wild-type strain (Fig. 4C). No appreciable differences were found in terms of the fatty acid composition, except for the Km6 *eht1^–^* strain bearing the *ACC1* plasmid, which had more than 80% of its total fatty acids comprised of stearic acid (18:0), compared to ∼10 to 20% in the other strains (see Fig. S5 in the supplemental material).

10.1128/mBio.01410-18.5 FIG S5 Fatty acid composition of Km6-derived strains. Eight fatty acids were measured using GC-FID; no appreciable difference in composition was seen among the strains, except for mutant Km6 *eht1^*–*^* bearing the ACC1 overexpression plasmid. Download FIG S5, TIF file, 0.95 MB. Copyright © 2018 Cernak et al. 2018 Cernak et al. This content is distributed under the terms of the Creative Commons Attribution 4.0 International license.

### Breeding to isolate high-producing, thermotolerant, and transformable strains.

**FIG 5:**
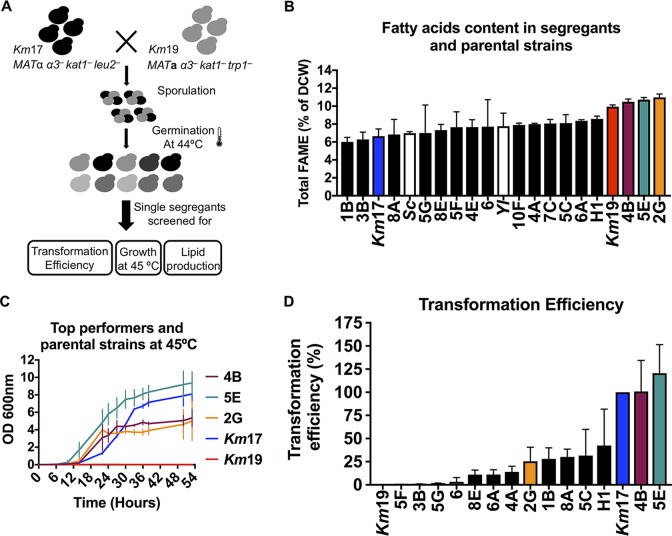
Selection of K. marxianus strains with combined beneficial traits. (A) Selection strategy. Km19 and Km17 were crossed, sporulated, and then germinated at 44°C to select for thermotolerant segregants. Single segregants were isolated and tested for lipid production, transformability, and high-temperature growth. (B) Fatty acid percentage in dry cell weight (DCW) for several segregants from the Km17 × Km19 cross and the parental strains. Three segregants have similar profiles to the more lipogenic parental strain (Km19). Experiments are from biological triplicates with mean and standard deviation shown. (C) Growth curves at 45°C for the segregants 4B, 5E, and 2G, as well as parental strains Km17 and Km19, in biological triplicate. Km19 is unable to grow at this temperature. Growth curves for parental strains at 30, 37, and 42°C can be found in the supplemental material (Fig. S7A), as well as for segregants at 30 and 37°C (Fig. S7B). (D) Transformation efficiency for several segregants normalized by Km17 transformation efficiency. Experiments are from 2 to 4 biological replicates with normalized mean and standard deviations shown.

The ability to cross phenotypically diverse and stable haploid K. marxianus strains should enable combining several beneficial traits into a single strain. For example, strain Km19 is the best lipid-producing strain we identified (Fig. 3A), but it is neither easily transformed nor thermotolerant compared to other K. marxianus strains. On the other hand, strain Km17 transforms easily and is thermotolerant (growing at 45°C) but is only moderately oleaginous (Fig. 3A). We therefore crossed these two strains to combine their beneficial traits into single isolates. We first engineered Km19 *α3^–^ kat1^–^ trp1^–^* using CRISPR_NHEJ_ and crossed a *MAT***a** isolate with an engineered stable haploid Km17 *MAT*α *α3^–^ kat1^–^ leu2^–^* strain. The resulting diploids were sporulated and then germinated at high temperature (44°C) to select for thermotolerant segregants, and 91 haploid progeny were picked to screen for lipid production and plasmid transformability (Fig. 5A).

Single segregants isolated from the above temperature selection were individually scored in terms of lipid production using Nile red staining and flow cytometry and displayed high variability in lipid production (see Fig. S6 in the supplemental material). A few segregants performed better than the parental Km19 haploid strain, but a number of these had high fluorescence due to aggregation, as determined by light microscopy, and were therefore excluded from further analysis. Strains that did not aggregate had their fatty acid percentage in dry cell weight and composition measured using gas chromatography-flame ionization detection (GC-FID) (Fig. 5B). Notably, three of these isolates produced lipids as well as the parent Km19 strain, while inheriting the thermotolerance and transformability of Km17 (Fig. 5C and D; see Fig. S7 in the supplemental material). These strains therefore combined all three beneficial traits of the parental strains.

10.1128/mBio.01410-18.6 FIG S6 Nile red staining and flow cytometry for each single segregant from the Km17 × Km19 cross. The diploids from this cross were sporulated, and spores were germinated at high temperature (44°C). Ninety-one spores were collected, grown under the lipogenesis condition, treated with Nile red, and subjected to flow cytometry. Download FIG S6, TIF file, 0.29 MB. Copyright © 2018 Cernak et al. 2018 Cernak et al. This content is distributed under the terms of the Creative Commons Attribution 4.0 International license.

10.1128/mBio.01410-18.7 FIG S7 Temperature dependence of the growth of K. marxianus strains tested for lipogenesis. (A) Growth curves of Km17 and Km19 in 50 ml YPD medium at 30, 37, 42, and 45°C and 250 rpm. Experiments are from biological triplicates. (B) Growth curves of the highly lipogenic isolates from mating Km17 and Km19. Cells were grown in 50 ml YPD medium at 250 rpm at 30 and 37°C. Experiments are from biological triplicates. Download FIG S7, TIF file, 0.80 MB. Copyright © 2018 Cernak et al. 2018 Cernak et al. This content is distributed under the terms of the Creative Commons Attribution 4.0 International license.

## DISCUSSION

Common metabolic engineering techniques are not ideal when dealing with complex phenotypes such as thermotolerance, productivity, and robustness (28). Therefore, agnostic approaches for combining complex traits into model yeast species are of high value, including directed evolution (29), genome-wide transcriptome engineering (30), and genome shuffling (31). However, these cannot substitute for classical sexual crossing for combining strain-specific traits. It is known that sexual reproduction enables adaptation to stressful industrial environments due to the faster unlinking of deleterious allelic pairs compared to clonal populations (10). Here we establish K. marxianus as a platform for synthetic biology by engineering stable heterothallic haploid strains that can be crossed to combine complex, unmapped multigenic traits into one strain.

In S. cerevisiae, inactivation of a single gene encoding HO endonuclease makes this yeast heterothallic and is sufficient to gain laboratory control of its mating cycle (32). While inactivation of the single transposase α3 can be used to cross K. marxianus strains (8), the resulting strains possess unstable mating types due to the presence of Kat1 and could randomly switch from *MAT***a** to *MAT*α (Fig. 1B and Fig. 2C; Fig. S3). To establish stable crossing in K. marxianus and abolish self-mating, we used CRISPR_NHEJ_ to inactivate both transposases that are responsible for mating-type switching (α3 and Kat1) (Fig. 1B). By simultaneously inactivating *ALPHA3* and *KAT1*, we created stable heterothallic *α3^–^ kat1^–^* strains that cannot switch mating type and, therefore, can be mated in a controlled manner with strains of the opposite mating type (Fig. 2A; Fig. S3). Alternative methods for creating stable haploids by deleting the silenced *MAT* loci have been used in yeast (33), but this strategy creates sterile strains and can be lethal if the endonuclease that initiates the double-strand break required for mating-type switching is not inactivated as well (34). Our strategy of inactivating both the α3 and Kat1 transposases preserves the ability to mate K. marxianus strains, an essential tool for synthetic biology, and to take advantage of this yeast’s remarkable phenotypic diversity. We successfully isolated heterothallic haploid strains from 12 wild-type isolates. These strains readily mate with each other, resulting in sporulation-competent diploids that segregate to viable haploid spores. Combined with CRISPR-Cas9 genome editing (Fig. 1) (6–9), these results establish a full set of tools for use of K. marxianus as a synthetic biology host and for future exploration of its biology on a genome-wide scale.

Using both sets of synthetic biology tools described here, we sought to exploit the diversity of K. marxianus as a thermotolerant, fast-growing, Crabtree-negative yeast. Screening 11 of the wild-type isolates for high levels of lipogenesis, we found high strain-to-strain variability in lipid production. Notably, strain Km19 produced ∼10% lipid by dry cell weight after 24 h at 42°C, a considerably shorter time than the 120 h required by wild-type Y. lipolytica to accumulate a similar amount of lipid (25). We find Km19 stores the vast majority of lipid as free fatty acids (FFAs) (Fig. 3C), in contrast to Y. lipolytica, which stores lipids as triacylglycerols (22, 35). FFAs are particularly suitable for the production of alkanes/alkenes and fatty alcohols, two types of high-value chemicals (36, 37). Although Km19 produces lipids at a high rate, it is not thermotolerant compared to other K. marxianus isolates (Fig. 5C) and is difficult to transform with plasmid DNA (Fig. 5D). To eliminate these barriers to conducting genetic engineering in the oleaginous strain Km19, we crossed it with Km17, which transforms well and grows well at 45°C. Notably, some progeny of this cross isolated as single segregants produced lipids to the same level of the parent Km19 strain while retaining thermotolerance and transformability of Km17 and were also not prone to aggregation. The power of combining multiple complex and valuable traits using stable heterothallic strains, together with CRISPR-Cas9 genome engineering, opens a new frontier to use K. marxianus as both a thermotolerant model species and an industrially relevant host.

## MATERIALS AND METHODS

### Strains, media, and culture conditions.

The K. marxianus strains used in this study were purchased from ATCC (American Type Culture Collection) or CBS (The Dutch Centraalbureau voor Schimmelcultures, Fungal Biodiversity Centre) or were obtained from an in-house collection. A complete list of all the wild-type strains is given in Table 1. Strains Km1, Km20, and Km21 have internal identity codes YST31, 1S300000, and 1S1600000, respectively. Strains were stored at −80°C in 25% glycerol. All experiments began by inoculation of a 12-ml culture tube containing 3 ml yeast extract-peptone-dextrose (YPD) or synthetic complete dextrose (SCD) medium with a single colony grown on a YPD medium agar plate. Cultures were shaken at 250 rpm. YPD agar consisted of 10 g/liter yeast extract, 20 g/liter peptone, 20 g/liter glucose, and 20 g/liter agar. SCD consisted of 2 g/liter yeast nitrogen base (YNB) without amino acids or ammonium sulfate, 1 g/liter complete supplement medium (CSM), and 5 g/liter (NH_4_)_2_SO_4_. Five percent malt extract medium was made by mixing 30 g of malt extract with 20 g of agar and bringing the volume to 1 liter with H_2_O and then was sterilized by autoclaving at 10 lb/in^2^ for 15 min. Sporulation (SPO) medium was made with 10 g/liter potassium acetate, 1 g/liter Bacto yeast extract, and 0.5 g/liter glucose. 5-FOA plates contained 2 g/liter yeast nitrogen base without amino acids or ammonium sulfate, 5 g/liter (NH_4_)_2_SO_4_, 1 g/liter complete CSM, 20 g/liter glucose, 20 g/liter agar, and 1 g/liter 5-fluoroorotic acid (5-FOA). Lipogenesis medium contained 2 g/liter YNB without amino acids and ammonium sulfate, 1 g/liter ammonium sulfate or monosodium glutamate, and 8% glucose or cellobiose.

### Genome sequencing and annotation.

A single-colony isolate of strain YST31 (Km1 [Table 1]) grown on a YPD plate was used to inoculate a YPD liquid culture and prepare genomic DNA using the YeaStar genomic DNA kit (Zymo Research). We submitted ∼5 mg of genomic DNA for small insert library preparation (∼250 bp) and Illumina sequencing. Library preparation and genome sequencing (Illumina HiSeq 2500) were performed by the UC Davis Genome Center DNA Technologies Core (http://dnatech.genomecenter.ucdavis.edu/). For YST31, we obtained 14,790,917 PE100 paired-end reads, and after trimming, we assembled reads into 116 scaffolds using CLC Genomics Workbench version 7.5.1. Default settings were used for quality trimming and *de novo* assembly. The median coverage was 250-fold, and the total genome assembly was 10,784,526 bp. Genome annotation was performed using an automated software pipeline, FGENESH++ (http://www.softberry.com) version 3.1.1. Genes were first predicted *ab initio* using FGENESH and then refined based on protein homology (38, 39). A custom BLAST database based on GenBank nr (downloaded 31 October 2014) was used for homology refinement of gene models. Gene prediction parameters were obtained from Softberry and were based on Saccharomyces cerevisiae gene models as the training set. The resulting annotation output files were renumbered and converted into GenBank format using custom scripts provided by Softberry.

### Cas9 plasmid construction.

To manipulate Kluyveromyces marxianus, we created a plasmid that can replicate in both Escherichia coli and K. marxianus and confers resistance to kanamycin and Geneticin, respectively. We used plasmid pOR1.1 (13), which can replicate in E. coli and S. cerevisiae, as a backbone for further manipulation. We identified and cloned an autonomous replicating sequence (ARS) from commercially available K. marxianus strain ATCC 36907 (Km11 [Table 1]) as follows. Using a YeaStar genomic DNA extraction kit (Zymo Research, D2002), genomic DNA was extracted from K. marxianus ATCC 36907. One microgram of genomic DNA was incubated with restriction enzyme EcoRI (NEB, R0101S) to fragment the DNA. In parallel, the S. cerevisiae 2μ origin of replication was replaced with an EcoRI digestion site in pOR1.1. The plasmid was then linearized with EcoRI and treated with shrimp alkaline phosphatase (Affymetrix, 78390) to dephosphorylate the DNA ends and prevent religation of the vector. The genomic DNA fragment pool was ligated with the linearized plasmid using T4 DNA ligase (Invitrogen, 15224017), transformed into One Shot TOP10 competent E. coli (C404003), and plated on kanamycin selection plates. All growing colonies were pooled, and the plasmids were extracted using the QIAprep spin miniprep kit (Qiagen, 27106). Two micrograms of the resultant plasmid pool was transformed into ATCC 36907 and plated on Geneticin selection plates. Many colonies were picked, and plasmid extraction was performed for each using the Zymo Research yeast plasmid extraction kit (D2001). The plasmids were individually transformed back into One Shot TOP10 competent E. coli, and the plasmids were extracted once more and digested with EcoRI. The digests were run on a 1% agarose gel with TAE (Tris-acetate-EDTA) buffer (40), and the clone with the smallest insert was chosen. The insert was sequenced and then systematically trimmed to a 232-bp functional region that still conferred the ability of the plasmid to replicate in K. marxianus (Table S2).

The resulting plasmid with a K. marxianus ARS was then modified to express Cas9 and a single-guide RNA (sgRNA) cassette using transcription promoters and terminators from K. marxianus. The S. cerevisiae promoter and terminator for Cas9 as used in pCas (13) were replaced with those for homologous genes in K. marxianus. In the new plasmid, Cas9 expression was driven by the promoter region of the gene *KmRNR2*—a mild-strength promoter—and terminated by the strong *KmCYC1* terminator. S. cerevisiae tRNAs were used as promoters to drive sgRNA expression (13) and terminated by the S. cerevisiae *SNR52* (*ScSNR52*) terminator. Between the promoter and the sgRNA, there is a hepatitis δ-ribozyme sequence that cleaves off the 5' leader sequence, liberating the tRNA from the sgRNA body that binds to Cas9 protein. The released transcript contains the δ-ribozyme, the protospacer sequence that targets Cas9 to the desired sequence, and the scaffold sgRNA (13).

### Overexpression plasmid construction.

Four genes found to be involved in lipogenesis in other yeasts were cloned into overexpression plasmid backbones using the In-Fusion cloning kit (Takara). The cloning reaction mixtures contained 25 to 50 ng of vector, 3 times molar excess of PCR-generated insert, and 0.5 μl of In-Fusion in a final volume of 2.5 μl. K. marxianus *ACC1* and *DGA1* coding sequences were amplified from Km6 gDNA using Phusion polymerase and cloned into two different linearized backbones, while Yarrowia lipolytica *ACL1* and *ACL2* coding sequences were cloned into the same plasmid. The vector backbone was the same used for pKCas9 construction, containing a K. marxianus ARS isolated as described above, a Geneticin resistance marker, and the pUC bacterial origin of replication. *ACC1* and *ACL1* were controlled by the K. marxianus GK1 promoter, while *DGA1* and *ACL2* were under the control of the *TDH3* promoter (Table S2). All ORFs were terminated by the K. marxianus *CYC1* terminator sequence. The resulting plasmids were transformed into wild-type K. marxianus strain Km6 (Table 1), as well as into 10 knockout mutant strains derived from Km6 that were constructed using CRISPR_NHEJ_: *adh5*^–^, *adh6*^–^, *adh4*^–^, *gpp1*^–^, *atf1*^–^, *eht1*^–^, *mfe1*^–^ *gpp1*^–^, *mfe1*^–^ *adh5*^–^, *pex10*^–^ *gpp1*^–^, and *pex10*^–^ *adh5*^–^ (Table S1).

### High-efficiency DNA transformation.

We established a high-efficiency transformation protocol for K. marxianus as follows. A single colony was inoculated in 1.5 ml of YPD medium and incubated at 30ºC overnight, then 180 μl of this culture was transferred to 5 ml of fresh YPD medium and incubated at 30ºC until the optical density at 600 nm (OD_600_) reached 1.0 to 1.2 (∼5 to 6 h). Then, 1.4 ml of the culture was aliquoted into microcentrifuge tubes and spun down at 3,000 × *g* for 5 min. The supernatant was removed, and the pellet was resuspended in 50 mM lithium acetate, followed by incubation at room temperature for 15 min. The cells were spun down, the supernatant was discarded, and the cells were used for subsequent transformation reactions.

Single-stranded DNA (ssDNA) was previously prepared as follows (41): 2 μg/μl of ssDNA from Sigma (D1626-250mg) was agitated with a stir bar overnight in TE buffer (10 mM Tris [pH 8.0] and 1 mM EDTA) at 4ºC and then concentrated to 10 μg/μl by isopropanol precipitation, resuspended in water, and quantified (NanoDrop 1000; Thermo Scientific). Prior to each transformation, aliquots of ssDNA were boiled for 5 min and then placed in an ice bath for 5 min. Keeping all the reagents on ice, 66.7 μl of 60% polyethylene glycol (PEG) 2050, 12.5 μl of 2 M lithium acetate, and water in a final volume of 100 μl were added to a sterile microcentrifuge tube. Then 2 μl of 1 M dithiothreitol (DTT) and 25 μg of ssDNA were added, followed by 0.1 to 5 μg of pKCas plasmid DNA. The transformation mixture was briefly vortexed, and 100 μl was added to the cells. The transformation reaction mixture was then incubated at 42ºC for 40 min. The reaction was spun down for 5 min at 3,000 × *g*, the supernatant was removed, and 500 μl of fresh YPD was added. The cells were allowed to recover for 2 h at 37ºC and 250 rpm. Then 10% of the volume was spread on a YPD G418 selection plate, and the remaining volume was spread on a second G418 plate.

### CRISPR_NHEJ_ and CRISPR_HDR_.

We transformed the pKCas plasmid into K. marxianus strains in the absence of donor repair DNA to determine the efficiency of NHEJ repair of the double-strand break. We cloned the guide sequence for the sgRNA in pKCas to target *URA3* (Table S2), to allow counterselection with 5-fluoroorotic acid (5-FOA) plates, which select for *ura3*^–^ colonies. Approximately 1 μg of pKCas plasmid was transformed into K. marxianus, and the efficiency of editing was calculated by counting the number of *ura3*^–^ colonies divided by the number of G418^R^ transformants. Sequencing of the targeted region revealed small insertions or deletions (indels) around the Cas9 cleavage site typical of NHEJ, resulting in premature stop codons within the *URA3* ORF (Fig. S1). We find that this system can be used to create inactive alleles in different genes with efficiencies of ∼75% (Fig. S1).

We modified the high-efficiency transformation protocol to enable cotransformation of the pKCas plasmid and a linear repair DNA template (donor DNA), for HDR-mediated genome editing. For tests of HDR-mediated gene insertion, the donor DNA targeted for genome integration contained the NatMX cassette conferring resistance to nourseothricin and 0.9 kb of flanking homology to the target site in the K. marxianus genome. Donor DNA was generated by PCR and concentrated by isopropanol precipitation (42). The best ratio of plasmid to donor DNA was 0.2 μg of pKCas plasmid and 5 μg of linear donor DNA, with 0.9 kb of homology to the Cas9 targeting site. Tests with higher concentrations of both pKCas and donor DNA were not as efficient. The transformation reaction was carried out as described above, except that cells were allowed to recover for 1 h at 37ºC in YPD without drug at 250 rpm, after which G418 was added and the cells were allowed to recover overnight. For the nourseothricin gene insertion experiments, cells were plated on YPD G418 plates, incubated at 37°C overnight, and then replica plated on either 5-FOA or nourseothricin plates, to identify Nat^R^ colonies with correct insertion in the *URA3* locus. Colony PCR performed on select Nat^R^ colonies (targeting outside the 0.9-kb homology arms of the NatMX cassette) confirmed NatMX cassette integration at the *URA3* locus.

### Mating-competent heterothallic strains.

*ALPHA3* and *KAT1* double-inactivation strains (α*3*^–^ *kat1*^–^) were constructed using CRISPR_NHEJ_ (Table S1). K. marxianus was transformed with either *KAT1-* or *ALPHA3*-targeting pKCas plasmids. Genomic DNA was isolated from G418^R^ colonies, the *KAT1* or *ALPHA3* regions were PCR amplified, and the PCR products were sequenced. Colonies with sequences containing indels that generated early stop codons were chosen and were saved as glycerol stocks. Single-inactivation strains were subjected to a second round of CRISPR_NHEJ_ to inactivate the second transposase, creating the α*3*^–^ *kat1*^–^ double mutants. These double mutants were then subjected to a third round of CRISPR_NHEJ_ targeting the *LEU2* or *TRP1* genes to create auxotrophic strains. The resulting strains were tested for mating type and heterothallic status by using a pheromone assay described below or by crossing them with reference heterothallic haploid strains, allowing haploid heterothallic *MAT***a** or *MAT*α strains to be successfully isolated. Some double-transposase-inactivated strains did not mate with the reference strains, possibly because these are stable diploids or triploids (12) or possibly due to chromosomal rearrangements (43). Although we performed the gene inactivations individually, we later tested and verified that simultaneous targeting of both transposases with CRISPR_NHEJ_ works efficiently in K. marxianus.

### Mating pheromone response assay.

We used reciprocal BLASTp using S. cerevisiae and K. lactis protein sequences as queries to identify two putative *MFA* genes (*KmMFA1* and *KmMFA2*), as well as the *MF*α gene (16, 17). The sequences in S. cerevisiae are encoded by genes YPL187W and YGL089C in the Saccharomyces Genome Database (44), and those in K. lactis are encoded by GenBank entry CAG99901.1 (RefSeq ID XP_454814.1) and as described in reference 17. To the best of our knowledge, these pheromones had not been previously identified in K. marxianus. We found two putative *MFA* genes (*KmMFA1* and *KmMFA2*) as well as the *MF*α gene (*KmMF*α*1*, which encodes 2 isotypes, *Km*MFα1 and *Km*MFα2) by reciprocal BLASTp using the S. cerevisiae and K. lactis protein sequences as queries (Table S2). Interestingly, the deduced **a**-factor amino acid sequences of *Km*MFa1 and *Km*MFa2 are not completely conserved, differing by 1 amino acid (Fig. 1C). Sequencing of *KmMFA1*, *KmMFA2*, and *KmMF*α from 8 unique strains shows full strain-to-strain conservation. Notably, *Km*MFa1 is completely conserved with the respective K. lactis sequence, suggesting a relatively conserved sexual cycle.

Synthetic **a**- and α-pheromones were obtained from Genemed Synthesis, Inc., and resuspended in water. To verify if the putative α-factors (*Km*Mfα1 and *Km*Mfα2) are viable and induce mating projections (“shmoo”) in engineered heterothallic cells, we performed a pheromone morphological response assay. Cells were grown in YPD medium to a density of 10^6^ cells/100 ml, and both pheromones were added to a final concentration of 25 mg/ml. Cells were then examined under the light microscope at different times. Mating projections were observed after 6 h for the mature α-factors *Km*Mfα1 and *Km*Mfα2, while no morphological differences were seen in the absence of synthetic pheromone. Strains sensitive to the α-pheromones were classified as putative *MAT***a**, and strains that exhibited no mating projections in the presence of either α-pheromone for 12 h were presumed to be stable *MAT*α haploids or *MAT***a**/*MAT*α diploid strains. The mating type of heterothallic strains was verified by sequencing of the *MAT* loci, by PCR amplification of the *MAT* loci with primers flanking the *MAT* loci, and by *MAT*-specific primers (Table S2 and Fig. S2). The same procedure was done using the synthesized **a**-factors, but they failed to induce mating projections in the strains tested, possibly because **a**-factors are reported to be heavily posttranslationally modified, unlike α-factors (45).

### K. marxianus auxotrophic mating.

Auxotrophic double mutant strains (α*3*^–^ *kat1*^–^) of either mating type were grown up overnight from a single colony in 5 ml SCD medium. Strains were pelleted at 3,500 × *g* for 5 min, followed by washing with 1 ml of sterile water. Strains were pelleted again and resuspended in 50 μl of water. Four microliters of the cell suspension was dispensed as a single drop (patched) onto 2% glucose plates or MA5 plates and allowed to dry. Strains to be mated containing a complementary auxotrophic marker (either *leu2*^–^ or *trp1*^–^) were dispensed on top of previous dried spots and allowed to mate at room temperature for 24 to 48 h. Mating plates were replica plated onto SCD agar plates minus both leucine and tryptophan. Diploid cells were grown at 30°C for 48 h. Freezer stocks were made by scraping off the diploid patch, resuspending in 25% glycerol, and freezing at −80°C.

### Sporulation and spore purification.

For sporulation, diploid strains were taken from freezer stocks and streaked onto an SCD agar plate minus leucine and tryptophan to ensure diploid strain growth. Single colonies were inoculated in 5 ml SCD liquid medium and grown overnight at 30°C. Many strains (including Km17) were observed to be unstable as diploids, and this treatment alone resulted in 25 to 90% sporulation. Strains or crosses that resulted in relatively stable diploids were then patched onto MA5 or SPO plates or suspended in 1 ml of M5 or SPO liquid medium and incubated at room temperature. Cells were observed under the optical microscope every 24 h for sporulation. Typically, sporulation was rapid and reached 25 to 95% sporulation in 24 h. However, recalcitrant strains would require up to 3 to 4 days.

Spore purification was performed as described previously (46). Diploid cultures from sporulation medium (MA5 or SPO medium) were scraped from solid medium or centrifuged at 3,500 × *g* from liquid medium for 5 min, resuspended into softening buffer (10 mM DTT, 100 mM Tris-SO_4_ [pH 9.4]) at a cell/spore density of ∼1 × 10^8^/ml, and incubated for 10 min at 30°C. The spore/cell suspensions were centrifuged at 3,500 × *g* and suspended in spheroplasting buffer (2.1 M sorbitol, 10 mM potassium phosphate [pH 7.2]) to ∼3 × 10^8^ cell spores/ml. Zymolyase-100T (United States Biological Corporation; Fisher Scientific) was added to a final concentration of 0.2 mg/ml, and the mixture was incubated at 30°C for 20 min. Spheroplasts and spores were centrifuged at 3,500 × *g* for 5 min, and spheroplasts were lysed by multiple washes with 0.5% Triton X-100.

### Complementation mating assay.

To further validate the role of α3 and Kat1 in switching mating types, we constructed expression plasmids containing the coding sequences of each protein to perform a complementation assay. The proteins were cloned into the same vector used for Cas9 expression, under the control of the moderate-strength promoter of the gene *KmRNR2*. Alternative plasmids bearing the native promoter of *KAT1* or *ALPHA3* were also constructed. These plasmids were transformed into α*3*^–^ *kat1*^–^ *leu2*^–^ strains of either mating type. To test transformants for mating-type switching, we crossed them with stable heterothallic reference strains of either *MAT***a** or *MAT*α mating types that also lack the ability to produce tryptophan (α*3*^–^ *kat1*^–^ *trp1*^–^). We inoculated 12 single transformants of each mating type in 20 ml of SCD medium and incubated them for 24 h at 30ºC and 220-rpm shaking. The cultures were then washed and resuspended in 70 μl of water. Four microliters of each cell concentrate was spotted onto glucose 2% agar plates to induce switching and, later, mating. Reference strains of the same and opposite mating type were spotted on top of the 12 spots of transformants. The plates were incubated for 24 or 48 h at room temperature and then replica plated onto SCD − (Leu, Trp) plates. Growth was only observed in spots where mating occurred.

### Culture conditions for inducing lipogenesis.

Single colonies were grown overnight in 3 ml of YPD medium at 30ºC and 225 rpm. Then 20 ml of YPD medium was inoculated with 0.5 ml of the overnight culture and grown at 30ºC and 225 rpm for 24 h. Cells were pelleted at 3,000 × *g* for 10 min, washed with 1 ml of water, and then transferred to 1.5-ml tubes before being pelleted again at 3,000 × *g* for 10 min. The pellet was resuspended in 1 ml of lipogenesis medium and transferred to a 12-ml round-bottom tube containing 3 ml of the same medium. For the strains containing overexpression plasmids, the lipogenesis medium had 1 g/liter of monosodium glutamate instead of ammonium sulfate due to Geneticin’s incompatibility with ammonium sulfate. Cultures were then shaken at 275 rpm at 42ºC for 24 h. Cells were harvested by transferring 250 μl of each culture to preweighed Eppendorf tubes, spinning down at 3,000 × *g* for 10 min, washing with 500 μl of water, and then resuspending the culture in 100 μl of water prior to freezing it in liquid nitrogen. Cells were kept at −80ºC prior to lyophilization. Culturing conditions for cellobiose-grown samples were similar, except that lipogenesis medium contained 8% cellobiose instead of glucose.

### Epifluorescence microscopy and flow cytometry.

To assay lipid production, K. marxianus cells were stained with the lipophilic dye Nile red (MP Biomedicals) which is permeable to yeast cells and a common indicator of intracellular lipid content. Single colonies of K. marxianus were grown in 3 ml of YPD medium overnight for at 30ºC with 250-rpm shaking. Cell concentrations were then normalized, inoculated in fresh lipogenesis medium, and grown for 24 h at 42ºC at 250 rpm. Cells were harvested by spinning down 1 OD_600_ unit at 3,000 × *g* for 3 min and then resuspended in 500 μl of phosphate-buffered saline (PBS) solution (Sigma Aldrich). Then 6 μl of 1 mM Nile red solution (in dimethyl sulfoxide [DMSO]) was added to the cells, and the mixture was incubated in the dark at room temperature for 15 min. Cells were spun down and washed in 800 μl of ice-cold water, spun down again, and resuspended in another 800 μl ice-cold water. Nile red-stained cells were examined on a Zeiss Axioskop 2 epifluorescence microscope at 553-nm excitation and 636-nm emission.

For flow cytometry, 300 μl cell solution was diluted in 1 ml of ice-cold water and tested in the BD Biosciences Fortessa X20 fluorescence-activated cell sorter (FACS) using a 25,000-cell count, a forward scatter of 250, a side scatter of 250, and the 535LP and 585/42BP filters for fluorescence detection. Fluorescence data were analyzed using FlowJo software (Tree Star, Inc., Ashland, OR), and mean fluorescence values were obtained.

### Isolation of high-lipid-producing strains.

Strains Km19 and Km17 were made heterothallic by CRISPR_NHEJ_ by inactivating both the *KAT1* and *ALPHA3* genes, as well as *TRP1* or *LEU2* (Table S1) to allow selection of diploids in SCD − (Leu, Trp) medium. Although Km19 does not transform well, by using our high-efficiency transformation protocol, we were able to recover a few colonies that transformed with the pKCas9 plasmid and allowed isolation of α*3*^–^ *kat1*^–^ auxotrophic strains. Km17 had a much higher transformation success rate, and triple-inactivation strains were easily constructed. To select for thermotolerant and lipogenic strains, Km19 *MAT***a** α*3*^–^ *kat1*^–^ *trp1*^–^ was crossed with Km17 *MAT*α α*3*^–^ *kat1*^–^ *leu2*^–^ on MA5 medium (Table S1). Diploid cells were sporulated, and a spore suspension was made. Spores were germinated in YPD liquid medium overnight at 30°C, plated onto YPD plates, and incubated at 44°C to select for thermotolerant strains. Ninety-one colonies were then selected and subjected to lipogenesis conditions for 24 h. The strains were ranked by Nile red mean fluorescence (Fig. S6), and those prone to flocculation as determined by light microscopy were eliminated from further analysis.

### Determination of fatty acids by gravimetric analysis and GC-FID.

To measure the percentage of fatty acids in dry cell weight, we extracted lipids from lyophilized cells and performed gravimetric analysis. Briefly, 250 μl of culture was pelleted at 4,000 × *g* for 10 min in preweighed 1.5-ml microcentrifuge tubes (Metter Toledo Excellence XS205DU balance). After the supernatant was removed, the cell pellet was suspended in 0.5 ml of water and pelleted again at 3,000 × *g* for 10 min, after which the pellet was resuspended in 100 μl of water. The samples were frozen and stored at −80ºC. Frozen samples were then lyophilized overnight and weighed to calculate dry cell weight.

To measure the percentage of fatty acids in the different strains, we extracted lipids from lyophilized cells prepared as described above and analyzed them by gas chromatography. Fatty acids were extracted and transesterified into fatty acid methyl esters (FAMEs) with methanol in the presence of an acid catalyst. The dry cell pellet was transferred to 15-ml glass conical screw-top centrifuge tubes, and 1 ml of methanolic HCl (3 N concentration) with 2% chloroform was added to the pellet. To ensure complete transesterification, an additional 2 ml of methanolic HCl (3 N) plus 2% chloroform was added. Approximately 100 μg of an internal standard (methyl tridecanoate) with its exact mass recorded was prepared in methanol and was added to the tube. The tube was sealed with a Teflon-coated screw cap and heated at 85°C for 1.5 h with vortexing every 15 min. The mixture was then cooled to room temperature, and the resulting FAMEs were extracted by the addition of 1 ml of hexanes followed by 30 s of vortexing. An organic top layer was obtained by centrifugation of the sample at 3,000 × *g* for 10 min. The top layer was carefully collected and transferred to a GC vial. One microliter was injected in split mode (1:10) onto an SP2330 capillary column (30 m by 0.25 mm by 0.2 µm; Supelco). An Agilent 7890A gas chromatograph equipped with a flame ionization detector was used for analysis with the following instrumental settings: injector temperature, 250°C; carrier gas, helium at 1 ml/min; and temperature program, 140°C, 3 min isocratic, 10°C/min to 220°C, 40°C/min to 240°C, and 2.5 min isocratic.

### Total oil extraction from dry yeast cells for thin-layer chromatography.

Total yeast oil was extracted following the protocol described by Folch et al (47) for analysis by thin-layer chromatography. Approximately 20 mg dry cell weight and 500 mg silica beads were weighed into a 2-ml centrifuge tube. One milliliter MeOH was added to the tube, and it was vortexed. Then, the tube was put into an aluminum block and bead-beaten 4 times for 30 s with 30-s resting intervals in between. The contents were transferred into a conical 15-ml glass centrifuge tube, and 0.25 ml MeOH was used to rinse the residuals on the small centrifuge tube. CHCl_3_ (2.5 ml) was added, and the tube was briefly vortexed. The tube was shaken for 1 h at 235 rpm. Two hundred fifty microliters of CHCl_3_-MeOH (2:1) and 1 ml of MgCl_2_ (aqueous) (0.034%) were added into the mixture, and the tube was shaken for 10 min. The solution was vortexed for 30 s and centrifuged at 3,000 rpm for 5 min, and the upper aqueous layer was removed. The resulting organic layer was washed with 1 ml 2 N KCl-methanol (4:1 vol/vol), vortexed, and centrifuged in the same way. The aqueous upper layer was removed, and the resulting organic layer was introduced with the artificial upper phase (chloroform-methanol-water at 3:48:47). The resulting mixture was vortexed and centrifuged, and the upper layer was aspirated. This step involving the artificial upper phase was repeated until the white layer at the interface completely disappeared.

### Thin-layer chromatography analysis of lipid composition.

TLC plates (7 by 10 cm) were preheated in a 120˚C oven for at least 10 min. A piece of paper towel/filter paper (7 by 10 cm) was added into a 600-ml beaker for saturation, and solvent system 1 (SS1: petroleum ether-Et_2_O-AcOH [70:30:2]) was added until the solvent reached about 0.5 to 1 cm in height. The beaker was covered with aluminum foil and Parafilm, and the resulting setup was left alone for at least 10 min. Compounds were spotted on the preheated TLC plate, and the plate ran in SS1 until the solvent front was halfway up the plate. The plate was dried at room temperature for 15 min. The plate was run using solvent system 2 (SS2: petroleum ether-Et_2_O [98:2]) until the solvent front nearly reached the top of the plate. The resulting TLC plate was dried under a fume hood for 30 min before being immersed in MnCl_2_ charring solution (0.63 g MnCl_2_⋅4H_2_O, 60 ml H_2_O, 60 ml MeOH, 4 ml concentrated H_2_SO_4_) for 10 s. The stained plate was developed in a 120˚C oven for approximately 20 min or until dark spots were observed.

### Data availability.

The raw fastq reads have been deposited in the NCBI SRA (accession no. SRP158013) and the scaffolds and annotation in Genbank under BioSample accession no. SAMN09839046.
