# Supplementary figures and images for: Engineering Kluyveromyces marxianus as a Robust Synthetic Biology Platform Host
Source: mBio. 2018 Sep 25;9(5):e01410-18. doi: 10.1128/mBio.01410-18 (PMC6156195; doi:10.1128/mBio.01410-18)

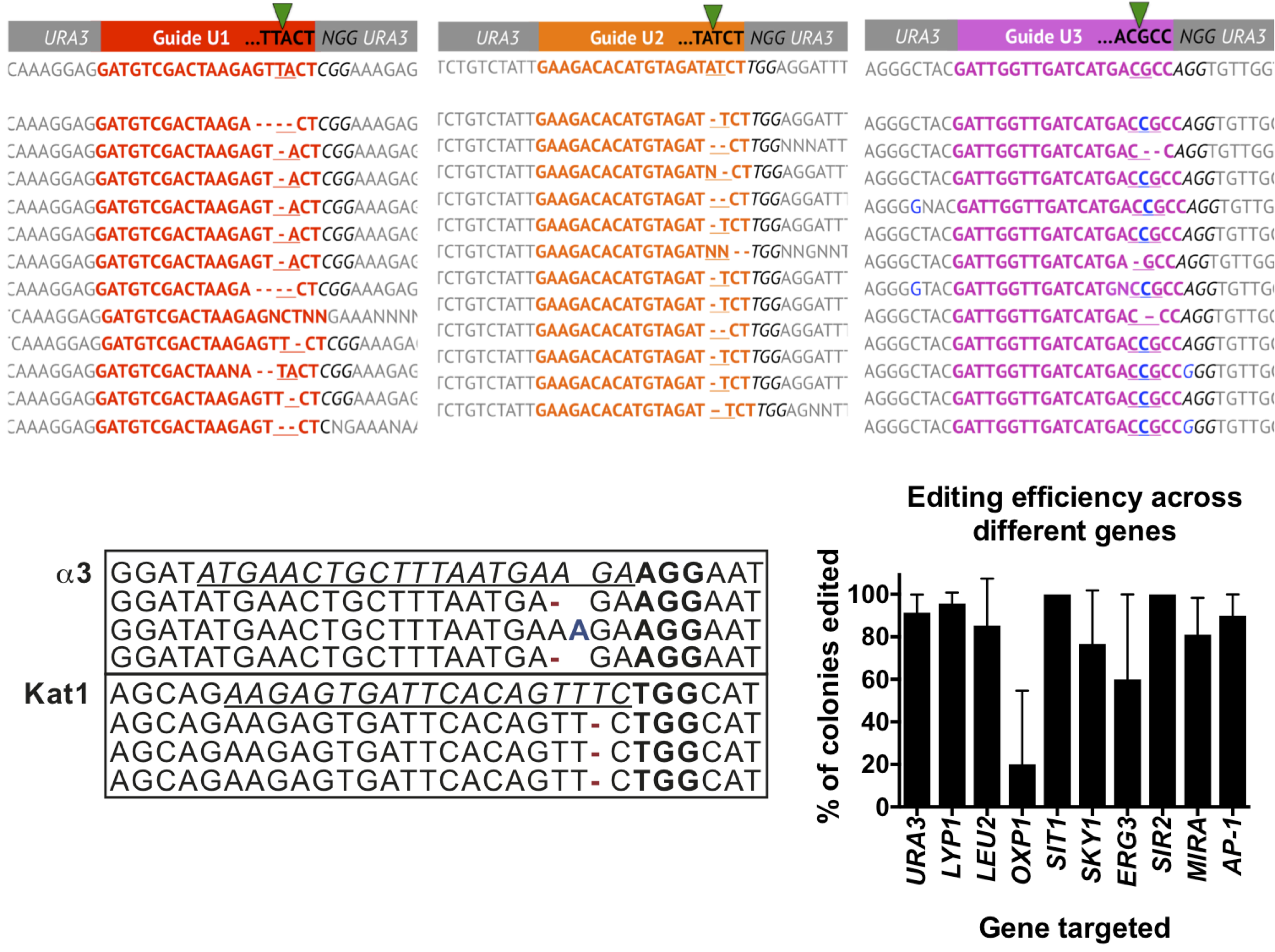

Supplement: FIG S1 [file mbo005184077sf1.tif]

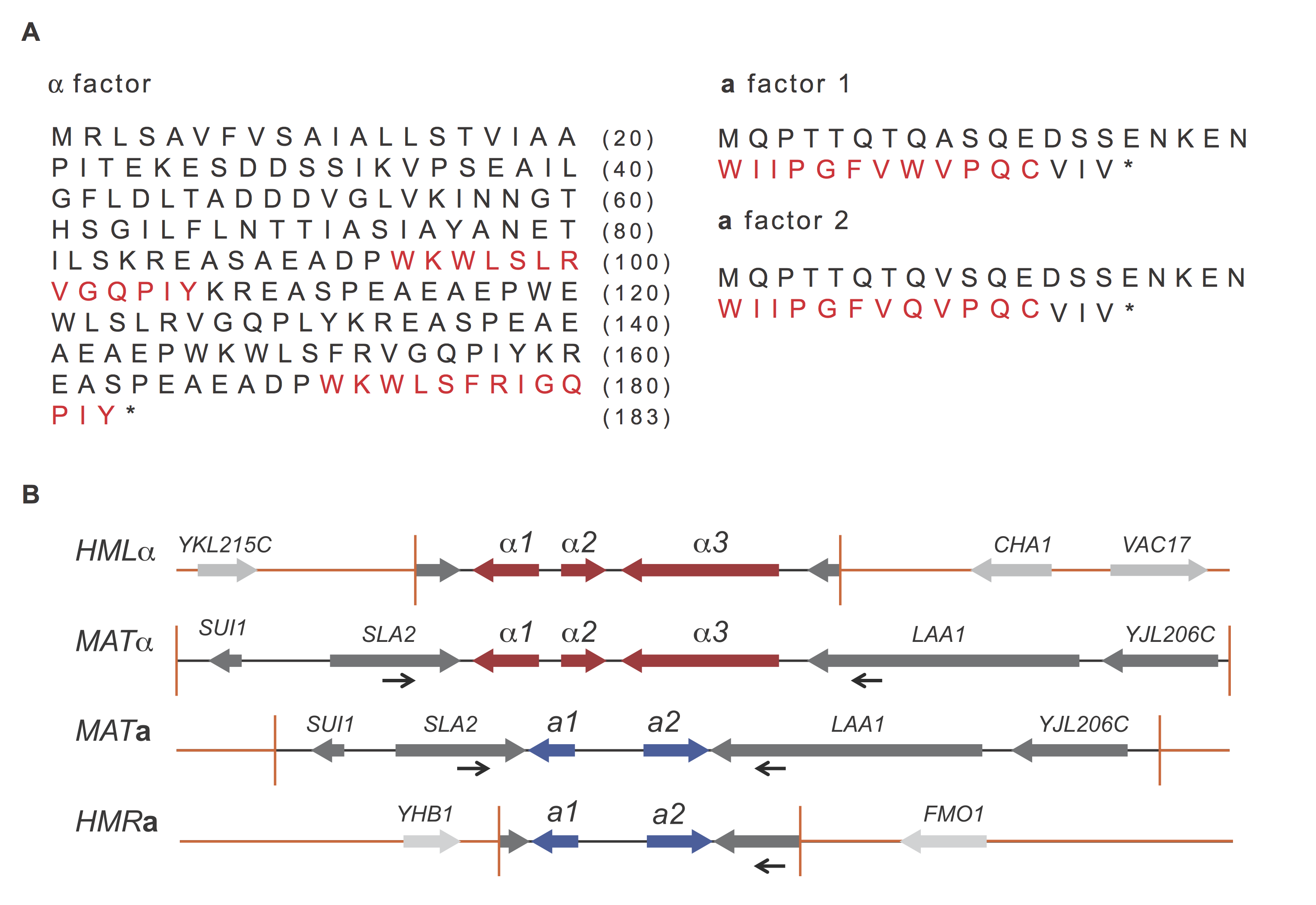

Supplement: FIG S2 [file mbo005184077sf2.tif]

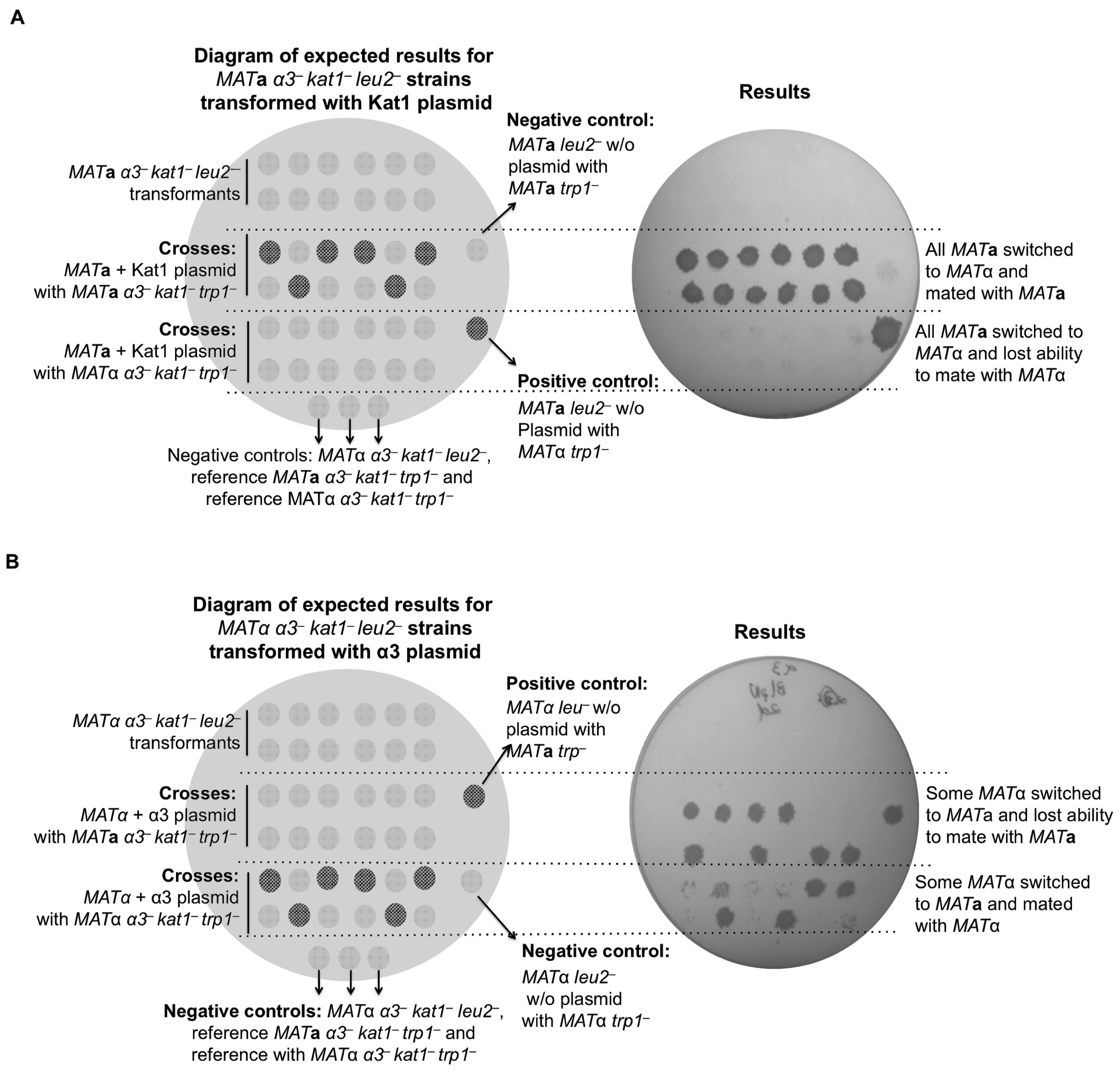

Supplement: FIG S3 [file mbo005184077sf3.tif]

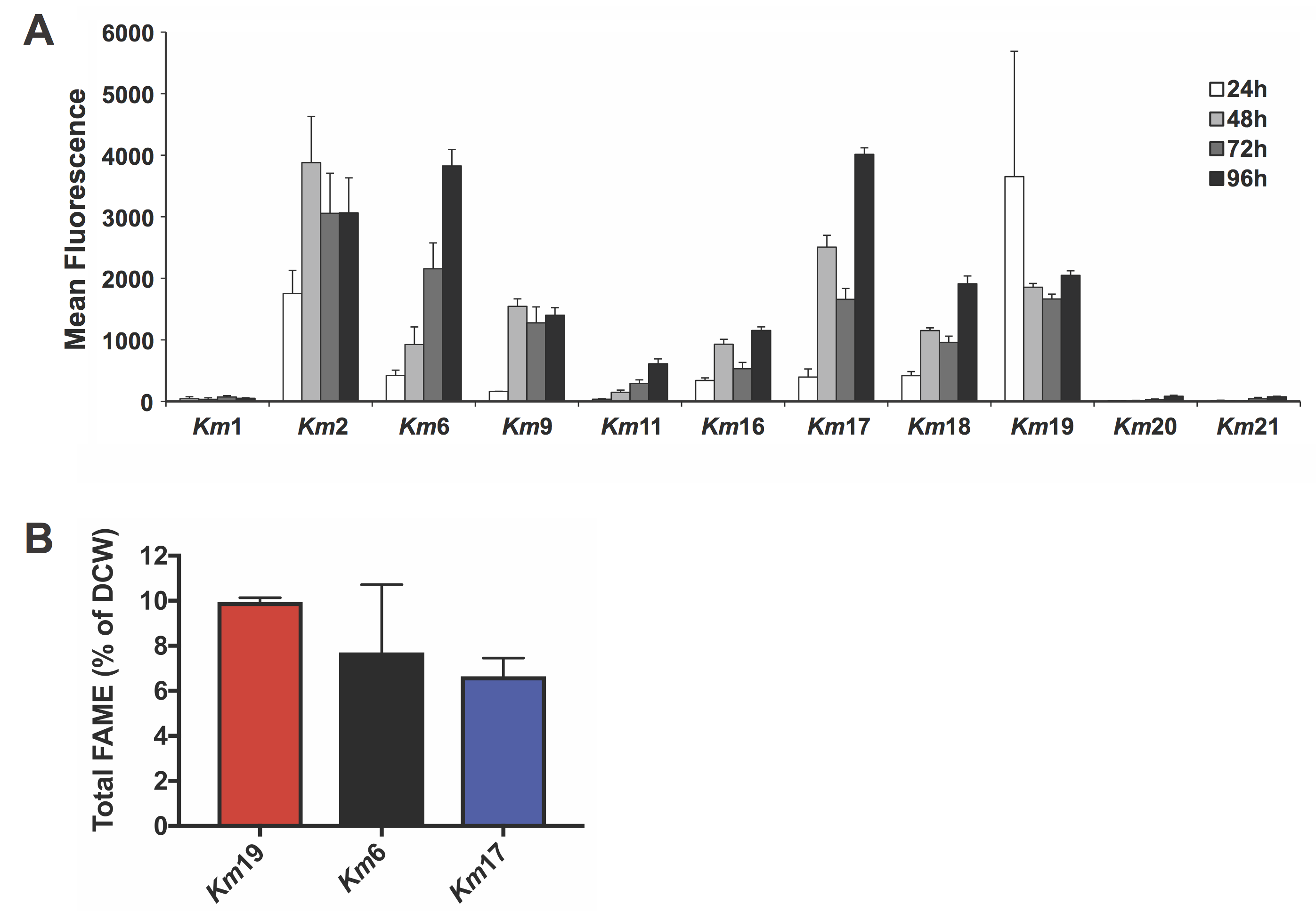

Supplement: FIG S4 [file mbo005184077sf4.tif]

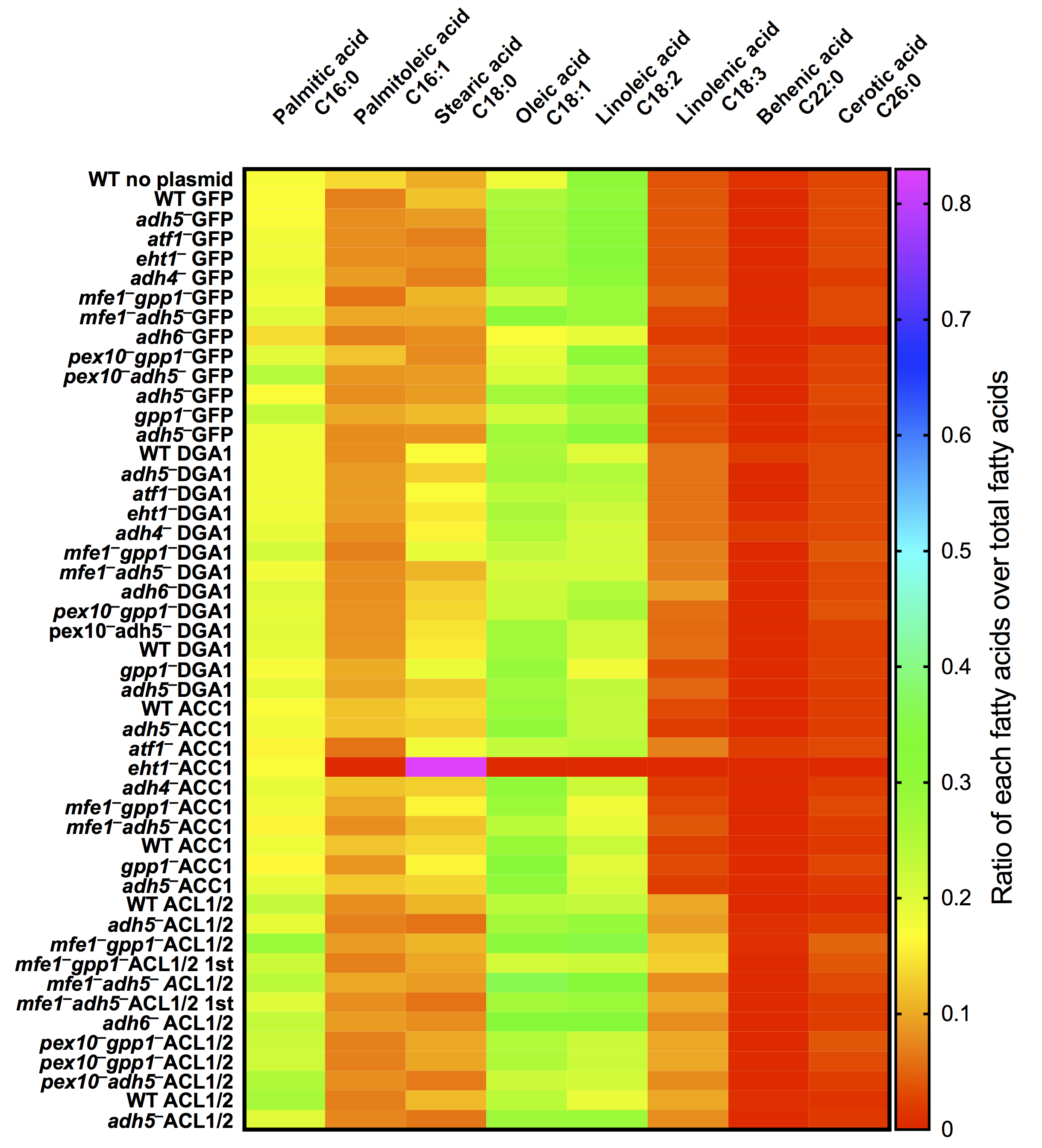

Supplement: FIG S5 [file mbo005184077sf5.tif]

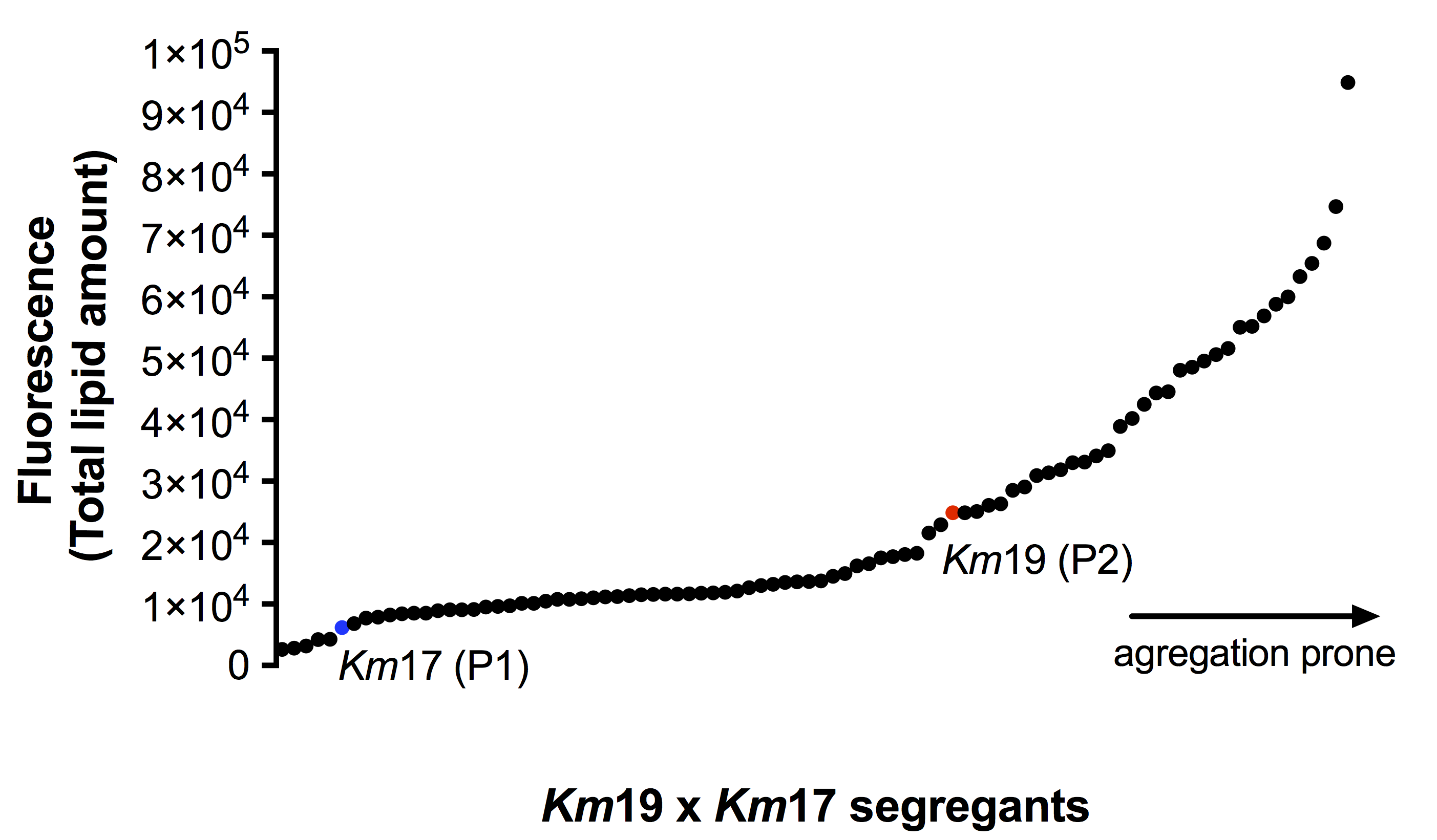

Supplement: FIG S6 [file mbo005184077sf6.tif]

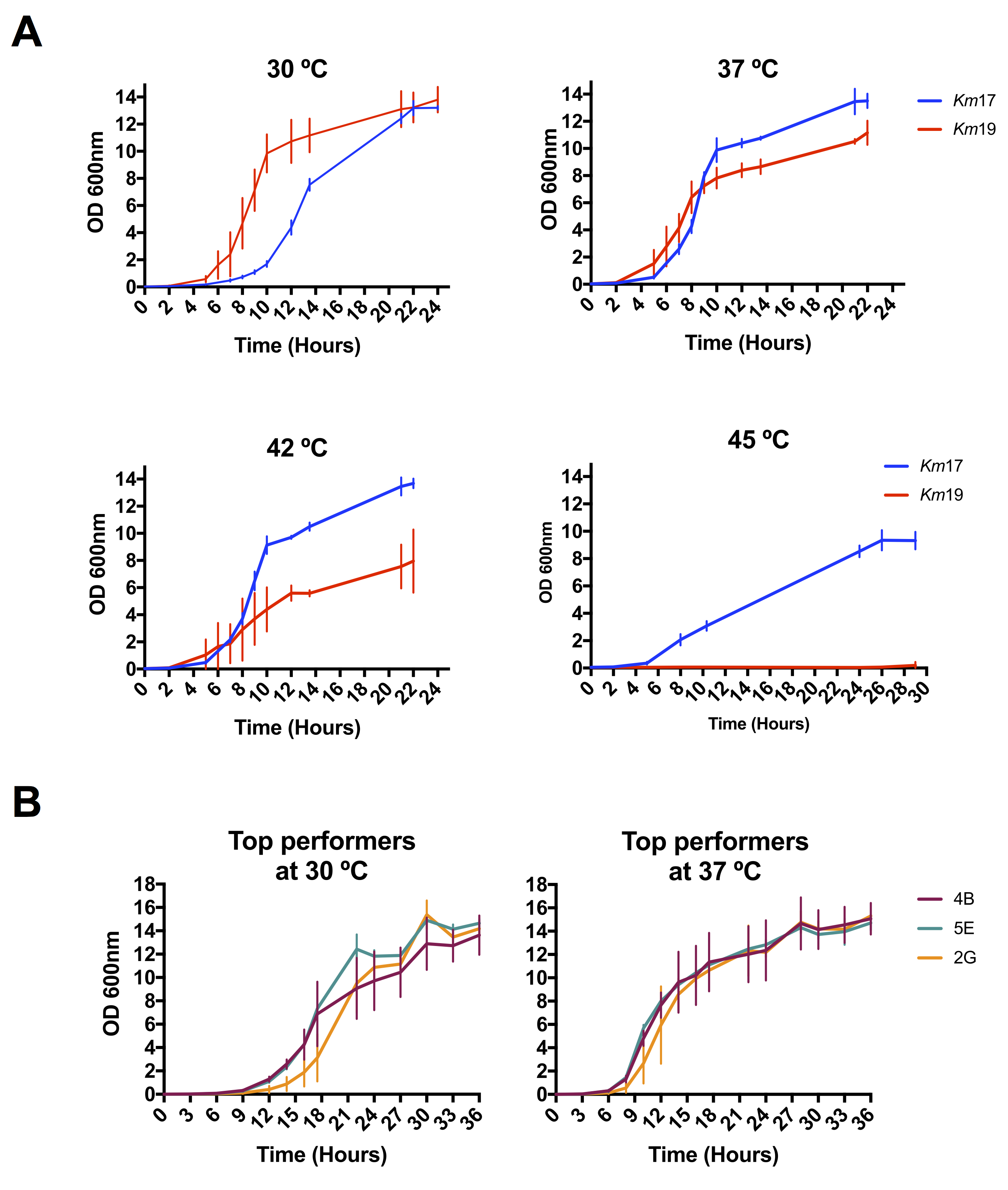

Supplement: FIG S7 [file mbo005184077sf7.tif]
